# Pension beneficiaries’ household food insecurity and associated factors in Debre Markos town, Northwest Ethiopia

**DOI:** 10.1186/s13104-018-3661-6

**Published:** 2018-08-06

**Authors:** Yawukal Chane, Getachew Hailu, Gemechu Kumera

**Affiliations:** 1 Department of Public Health, College of Medicine and Health Sciences, Hawassa University, Hawassa, Ethiopia; 2 Department of Public Health, College of Medicine and Health Sciences, Debre Markos University, Debre Markos, Ethiopia

**Keywords:** Household food insecurity, Pension beneficiaries, Northwest Ethiopia

## Abstract

**Objectives:**

A community based cross-sectional study was conducted from March to April, 2016 in Debre Markos town, Northwest Ethiopia to assess the level of household food insecurity and associated factors among pension beneficiaries.

**Results:**

The overall prevalence of household food insecurity among pension beneficiaries’ households was 82.5%. Among food insecure households, 4.9% were labelled as mildly, 48.5% moderately and 46.6% severely food insecure. Living in rental house (P = 0.05), being younger beneficiaries (P = 0.001), low monthly household income (P = 0.001) and poor self-reported health status (P = 0.03) were found significantly associated with household food insecurity. In conclusion, food insecurity was a public health problem among pension beneficiaries in the study area. The effort of the government to increase the pension benefit and making especial subsidy on food and health costs yield a long-term solution.

## Introduction

Food security is a broad concept which involves conditions related to food supply and demand. Food security is defined as a state in which “all people at all times, have physical and economic access to sufficient, safe and nutritious food to meet their dietary needs and food preferences for an active and healthy life” [1, 2]. Food insecurity (FI) refers to limited or uncertain availability of nutritionally adequate and safe foods, or limited or uncertain ability to acquire food in socially acceptable ways [3].

Various studies conducted over years have provided evidence that FI is associated with a broad spectrum of health consequences [4–10]. FI is associated with malnutrition due to inadequate nutrient intake [9–14]. Previous studies also reported an association of FI with mental health problems [15–17], psychosocial dysfunction in children [18], compromised health status in the elderly [19], and socio-familial problems [20] and poor quality of life [21, 22].

FI continues to be a major public health problem worldwide. Globally, over 800 million people do not eat enough food to live an active and healthy life [23, 24]. Therefore, FI is an ongoing public health issue in both developing and developed countries [25]. The vast majority of food insecured people live in developing countries [26, 27]. Ethiopia is among the countries with high prevalence of household food insecurity (HFI). According to a baseline national food security survey by the Ethiopian Health and Nutrition Research Institute (EHNRI), 35% of households in Ethiopia were food insecure [28]. Another study conducted in Addis Ababa reported that 75% of households were food insecure [29]. Studies conducted in Nigeria [30], Cape Town [31], Manitoba [32], Cambodia [33], India [34], Canada [35], and USA [36, 37] also reported high prevalence figures.

Despite the current inflation and high market instability in Ethiopia, pension beneficiaries have constant income of 30–70% of their monthly salary, since 2011 [38]. Despite this fact there was no such study which assessed the level of food insecurity among this vulnerable group in Ethiopia. The findings of this study contribute to the understanding of the factors at multiple spheres which are predictive of FI among the pension beneficiaries. Further, data on the level and factors associated with FI are needed for prioritizing, designing and initiating intervention programs aimed at improving food security status of the pension beneficiaries. Thus, the aim of this study was to provide information regarding the level and factors associated with household food insecurity among pension beneficiaries.

## Main text

### Methods

#### Study setting

A community based cross-sectional study design was employed to assess the level and associated factors of HFI among pension beneficiaries in Debre Markos town, Northwest Ethiopia from March to April, 2016. According to 2015 Population and Housing census, the population of Debre Markos town is projected to be 107,000 [39]. The study population was households with pension beneficiaries living in Debre Markos town.

#### Sample size and sampling technique

The sample size was determined using single population proportion sample size calculation formula with the inputs 95% confidence level, 5% of margin of error, non-response rate of 5% and a hypothesized proportion of HFI (P) 50%. The required total sample size was 403. Households with at least one member of pension beneficiary were identified using names of individuals in the registration lists of a monthly pension payment payroll matching with their respective house numbers, documented in pensioners and social security affairs office of Debra Markos town [40]. A systematic random sampling technique was used to select study households using the house numbers. The sampling interval (K) was considered to be 8 since the total pension beneficiary households in the town were 3220, and the required sample size was 403. Every 8th households of pension beneficiaries were included in the study, and the 4th listed household was identified as a random start with a lottery method for the subsequent selection until the required sample size of 403 was obtained.

#### Data collection procedures

A structured and pre-tested questionnaire was used for assessing potential determinants of FI. The parts of the questionnaire on HFI level was adopted from Food and Nutrition Technical Assistance (FANTA) indicator guide for Household Food Insecurity Access Scale (HFIAS) [41]. The questionnaire was administered in a local language (Amharic). The translated Amharic version (local language) was pre-tested prior to the actual survey and modifications were made accordingly. The content validity of the tool was checked against the conceptual framework of the study by relevant professionals. Reliability of the tool was checked using a test–retest method. Questions with less than 0.7 kappa or Pearson coefficient values were removed or revised.

#### Data processing and analysis

Data were entered into EPI INFO version 7 and analysed using SPSS version 20. Bivariate and multivariable logistic regression analysis were used to assess the relative effect of various explanatory variables on the dependent variable and to control potential confounders. Independent variables significantly associated with the dependent variable in bivariate regression models were exported to multivariable regression models for adjustment. The model adequacy was checked by using Hosmer and Lemeshow goodness of fit test. *P* value ≤ 0.05 at 95% CI was considered statistically significant.

#### Ethical consideration

Ethical clearance was obtained from ethical review committee of Debre Markos University prior to data collection. Verbal consent was obtained from each study participants prior to participation in the study after detailed explanations about the purpose, benefit and the possible risks of the study. Nutrition education was given to all study participants.

### Results

#### Socio-demographic characteristics of study participants

Table 1 summarizes socio-demographic characteristics of the study participants. Of 403 pension beneficiary households recruited, 395 were willing to take part in the study, with a response rate of 98%.

**Table 1:** Socio demographic characteristics of pension receivers (study participants) in Debre Markos town, Northwest Ethiopia, 2016

Characteristics	Frequency (n)	Percent (%)
Age (years)		
22–44	39	9.9
45–64	180	45.6
65–94	176	44.5
Gender		
Male	141	35.7
Female	254	64.3
Marital status		
Married	140	35.4
Not union	26	6.6
Widowed	229	58.0
Family size		
< 4	288	72.9
5–9	107	27.1
Educational status		
Cannot read and write	150	38.0
Can read and write	39	9.9
Primary school	65	16.4
Secondary school	83	21.0
Certificate and above	58	14.7
Occupation before retirement		
Military	53	32.7
Civil servant	109	67.3
Current occupation		
Employed	68	17.2
Merchant	24	6.1
Daily labourer	28	7.1
Jobless	275	69.6

#### Health status, reason for pension and service year of study participants

According to a self-reported health status, 23.3% (n = 92), 46.3% (n = 183) and 30.4% (120) of study participants reported good, fair and bad health status, respectively. Reason for pension benefit for majority, 59.2% (n = 234) of study participants was old age retirement pension. The median service year of study participants before retirement was 32 years, ranging between 2 and 55 years.

#### Household income and expenditures

The median monthly pension income was 350ETB (17.5 USD), which is equivalent to 0.58 US dollar per day. Nearly half of study participants, 46.3% (n = 183) had monthly household income less than 33.3 USD. The median monthly household income was 703 ETB (35.15 USD). Majority of the study participants, 78.3% (n = 311) expend more than 75% of their monthly income on food.

The monthly median expenditure of households on food was 700ETB (35 USD). More than one-third of the study participants, 34.9% (n = 138) live in rental house. The households which had gardening accounts for only 17.2% (n = 68) of the households sampled.

#### Level of household food insecurity and Household access to food

The overall prevalence of HFI among pension beneficiaries’ households was (82.5%**)**. According to the scale, 46.6% (n = 152) of households were classified as severely food insecure, while 48.5% (n = 158) and 4.9% (n = 16) of households were moderate and mildly food insecure, respectively. From a total household, only 19.2% (n = 76) have never worried having enough food in the last 4 weeks (Table 2).

**Table 2:** Frequency distribution of responses to HFIAS items of study participants in Debre Markos town, Northwest Ethiopia, 2016

Characteristics	Frequency (n)	Percent (%)
Worry not have enough foods		
Yes	319	80.8
No	76	19.2
Unable to eat preferred foods		
Yes	317	80.3
No	78	19.7
Eat a limited variety of foods		
Yes	312	79
No	85	21
Eat some foods didn’t want		
Yes	264	66.8
No	131	33.2
Eat fewer meals in a day		
Yes	208	52.7
No	187	47.3
Eat a smaller meal than needed		
Yes	258	65.3
No	137	34.7
No food to eat of any kind		
Yes	92	23.3
No	303	76.7
Go to sleep at night hungry		
Yes	88	22.3
No	307	77.7
Go a whole day and night without eating		
Yes	46	11.6
No	349	88.4

#### Factors associated with household food insecurity

The multivariable logistic regression analysis revealed that age, monthly household income, house ownership, expenditure on food share, and health status were predictors of household food insecurity (Table 3).

**Table 3:** Factors associated with household food insecurity among pension beneficiaries in Debre Markos town, Northwest Ethiopia, 2016

Predictors	Food insecurity		COR (95% CI)	AOR (95% CI)	P-value
	Yes	No			
Age					
22–44	9	86	1		
45–64	28	80	0.29 (0.13–0.65)	0.18 (0.06–0.50)	0.001
≥ 65	31	160	0.54 (0.25–19)	0.26 (0.10–0.70)	0.007
Monthly household income (USD)					
< 33.3	1	182	1	1	
33.3–112.1	38	125	0.018 (0.01–0.13)	0.026 (0.003–0.19)	0.001
> 112.1	30	19	0.003 (0.003)	0.006 (0.001–0.053)	0.001
Monthly food expenditure (%)					
< 75	34	50	1	1	
≥ 75	35	276	5.36 (3.06–9.39)	3.33 (1.56–7.12)	0.002
Housing ownership					
Own house	60	197	1	1	
Government	8	96	3.66 (1.68–7.95)	2.74 (1.06–7.10)	0.03
Private rent	1	33	10 (1.35–75.04)	8.27 (0.94–73.06)	0.05
Self-reported health status					
Good	26	66	1	1	
Fair	34	149	1.73 (0.96–3.11)	2.53 (1.15–5.57)	0.02
Poor	9	111	4.85 (2.15–11)	3.10 (1.14–8.45)	0.03

### Discussion

The current study provide evidence for the high level (82%) of food insecurity among pension beneficiaries in Debre Markos town, Northwest Ethiopia. The findings are considerably higher than the level of national food insecurity (35%) reported by the EHNRI [28]. The level of HFI reported in the current study is also higher than in previous study conducted in Addis Ababa (75%) [29]. The higher level of FI in the current study could be due to low monthly income which is obtained from pension, and most of beneficiaries were survivor pensioners and unable to work or have less access to employment. The high level of FI in the present study might be partially explained by high food inflation in the country during the past 2 years. Moreover, high dependency of households on food supplied by the market, and fluctuating food prices are contributing to higher FI among study participants.

Our findings illustrated a significant association between food insecurity and age of pension beneficiaries, such that older pension beneficiaries were less likely to be food insecure as compared to younger beneficiaries. Our findings are consistent with previous studies from the Andes [42], US, Canada and Australia [43, 44]. Possible explanations for these differences may be reliance on their son or daughter and spiritual or religious affiliations. Other possible explanation may be older beneficiaries may have less dependents and more mature families than younger, and mature families may be more likely to be food secure and economically stable because they have more adults to generate income. Furthermore, possibly older beneficiaries are no longer living independently in the community.

Low family monthly income was found to be negatively associated with food security. This finding is consistent with other studies conducted in Addis Ababa [29], Nigeria [30], Canada [32] and Australia [45, 46]. The study finding showed a positive impact of income on food security. This might be due to the reason that low monthly income diminishes the purchasing power of household consumption items. Further, income determines how much can be spent on various needs of the household.

Food share expenditure was the other predictor of FI in the study population. HH which expend more than 75% of their monthly income on food were at greater risk of being food insecured. Other studies in Bolivia, Burkina Faso, Philippines [47] and Manitoba [32] also reported parallel finding. The current study found that majority of the monthly income was spent on purchasing food and, even then, the prevalence of food insecurity was high among study participants. It might be due to the low income, where the amount spent on food may be insufficient to meet the food requirements of all the family members. This, in turn, may affect the food security status of the household. As the income decreases they intend to expend on food more than any other needs. Moreover, it might be partially explained by increasing price of food in the country during the past 2 years that led them to expend more of their income on food.

Households living in rental house were at greater risk of being food insecure as compared to those who had their own house. Our finding is consistent with previous research from Australia [46]. This might be due to the reason that pensioner who had their own house can rent some of their room and can earn income. It might be also due to the reason that the government rented house is given for more vulnerable pension beneficiaries.

Self-reported poor or fair health was an independent predictor of HFI. Previous studies also supported the finding [32, 42, 46, 48–50]. Poor health condition may limit physical access to food in terms of of going the market, get to the store and to purchase, lifting, preparing and cooking meals at home [51]. Further, poor health status may impact upon their income and expenditures due to their inability to work, thus impacting their food security. However, as this study was cross sectional, it is not possible to determine whether poor health is a risk factor or an outcome of food insecurity.

### Conclusion

The study findings provide evidence for the public health significance of food insecurity among pension beneficiaries’ household in the study area. Living in rental house, being younger beneficiaries, low monthly income and self-reported poor/fair health status are key predisposing factors to food insecurity. The study findings provide evidence for the public health significance of food insecurity among pension beneficiaries’ household in the study area. Living in rental house, being younger beneficiaries, low monthly income and self-reported poor/fair health status are key predisposing factors to food insecurity. Addressing the needs of the pension beneficiaries who are at greater risk of food insecurity will require a coordinated approach both nationally and at a local level. In order to mitigate this problem, the effort of the government to increase the pension benefit and making especial subsidy on food and health costs yield a long-term solution. Nutrition programs should recognize and provide services to cover pension beneficiaries needs. Social support and income generation strategies are also recommended. Ensuring employment opportunities will also have affirmative input. We also suggest national level study with strong study design.

## Limitations

The major limitation of this study was the cross-sectional nature of its design as we can’t establish causal relationships between the independent variables and household food insecurity. Secondly, recall bias and social desirability bias are potential limitations that may influence the HFIAS questions though attempt was made to minimize it by clarifying the purpose of the study. The health status was determined by using self report, which may not necessarily indicate their health status.

## Abbreviations

EHNRI: Ethiopian Health and Nutrition Research Institute
ETB: Ethiopian Birr
HH: house hold
FI: food insecurity
HDDS: household dietary diversity score
HFI: household food insecurity
HFIAS: Household Food Insecurity Access Scale
FANTA: Food and Nutrition Technical Assistance
